# The Maintenance of Telomere Length in CD28+ T Cells During T Lymphocyte Stimulation

**DOI:** 10.1038/s41598-017-05174-7

**Published:** 2017-07-28

**Authors:** Ejun (Elijah) Huang, Enzo Tedone, Ryan O’Hara, Crystal Cornelius, Tsung-Po Lai, Andrew Ludlow, Woodring E. Wright, Jerry W. Shay

**Affiliations:** Department of Cell Biology, UT Southwestern Medical Center, 5323 Harry Hines Blvd., Dallas, TX 75390 USA

## Abstract

Telomerase activity is not readily detected in resting human T lymphocytes, however upon antigen presentation, telomerase is transiently upregulated. Presently, it is not known if telomerase activation is necessary for the proliferation of T cells or for the maintenance of telomere lengths. In this study, we found that telomerase activation is not required for the short- term proliferation of T cells and that telomeres progressively shorten in a heterogeneous population of T cells, even if telomerase is detected. By measuring telomerase activity at the single-cell level using quantitative ddPCR techniques (ddTRAP) and by monitoring changes in the shortest telomeres with more sensitive telomere length measurement assays, we show that only a subset of CD28+ T-cells have robust telomerase activity upon stimulation and are capable of maintaining their telomere lengths during induced proliferation. The study of this T-cell subset may lead to a better understanding on how telomerase is regulated and functions in immune cells.

## Introduction

Telomeres are repeated DNA sequences (TTAGGG_n_) that in combination with 6 shelterin proteins cap the ends of chromosomes to prevent the telomeres from being recognized as DNA damage^1^. The end replication problem (failure of lagging strand DNA synthesis to be fully replicated) results in the loss of DNA at the telomeres after each round of cellular DNA replication^2^. As a result, all human somatic cell telomeres become progressively shorter as cells divide. Progressive telomere shortening during each cell division finally leads to one or more critically short telomeres, initiating a DNA damage response signal that is referred to as replicative senescence^3, 4^. Previous cross-sectional studies have shown progressive telomere shortening in human lymphocytes from different age groups from newborn to 90 years old individuals^5^. To compensate for telomere loss during cell division, some proliferating cells transiently express telomerase, a cellular reverse transcriptase that maintains telomeres by adding telomeric repeats to chromosome ends during DNA replication^1, 4^. Telomerase is a ribonucleoprotein enzyme complex having a role in several essential cell signaling pathways^6^. The functional telomerase holoenzyme consists of an essential reverse transcriptase (*hTERT*) component and a template RNA (*hTR/hTERC*) along with several other associated proteins and is constitutively expressed in 85–90% of malignant human tumors^7^. However, in normal cells and tissues, telomerase expression is highly regulated and restricted to some proliferating transiently amplifying stem cell compartments^8^.

It is known that upon mitogen stimulation, immune cells can be activated and divide rapidly. It is well established that during this process telomerase is transiently activated^9^. Since this earlier study, a few reports have investigated the relationship between telomerase activation, telomere dynamics, and cell proliferation capacity. For example, telomerase activity is inhibited by actinomycin D or cycloheximide^10^, indicating that RNA and protein synthesis are both required for telomerase activation. Other studies have shown that telomerase is not exclusively regulated at the transcriptional level in stimulated T cells, as *hTERT* post-translational phosphorylation and nuclear translocation are essential to promote telomerase activity^11, 12^. Although there appears to be a positive correlation between the magnitude of telomerase activity and the ability of T cells to respond to antigen-induced stimulation, it has been shown that *hTERT* knockdown does not affect the rate of T cell proliferation^13^. Furthermore, it has been shown that neither *hTERT* nor *hTR* knockdown induced increases in the rate of telomere shortening during T cell stimulation^13^. In contrast, *hTR* may also play an anti-apoptotic role in human immune cells that is independent of telomerase activity, while overexpression of *hTERT* protein may lead to apoptosis by depleting *hTR* ^13^. However, others have compared telomere lengths among heterogeneous populations of peripheral blood mononuclear cells (PBMCs) before and after stimulation and found lengthening of average telomeres after stimulation^14^.

The studies described above employed semi-quantitative assays to measure telomerase activity and average telomere length from different cell populations^10–14^. However, immune cells, especially T cell populations, consist of an extremely heterogeneous mixture of cell types^15^. Thus, results published on heterogeneous T cell populations might not reflect the dynamics occurring in specific T cell sub-fractions. Also, monitoring subtle telomere changes is challenging due to the lack of a sensitive assay for quantifying the length of the shortest telomeres in cells.

By taking advantage of novel techniques that can measure telomerase activity at the single-cell level using quantitative ddPCR techniques (ddTRAP)^16^ and by monitoring subtle telomere changes using limited DNA input and more sensitive telomere length measurement assays, in this report we show that only a subset of CD28+ T-cells show robust telomerase activity upon stimulation. This subset of T-cells appear to be capable of maintaining their telomere lengths whereasCD28- cells have significantly more short telomeres, even though they proliferate at similar rates compared to CD28+ cells. This work emphasizes the importance of identifying specific subsets of T cells in the study of telomere and telomerase dynamics and their relationship with immune responses. Investigating the function of telomerase activation during T cell stimulation may provide new insights into understanding normal immune responses (T cell *in vivo* proliferation), as well as T cell *ex vivo* expansion, which is a critical requirement for recent immunotherapy protocols.

## Results

### Transient telomerase activity levels in stimulated T lymphocytes are comparable with cancer cell lines

**Figure 1:**
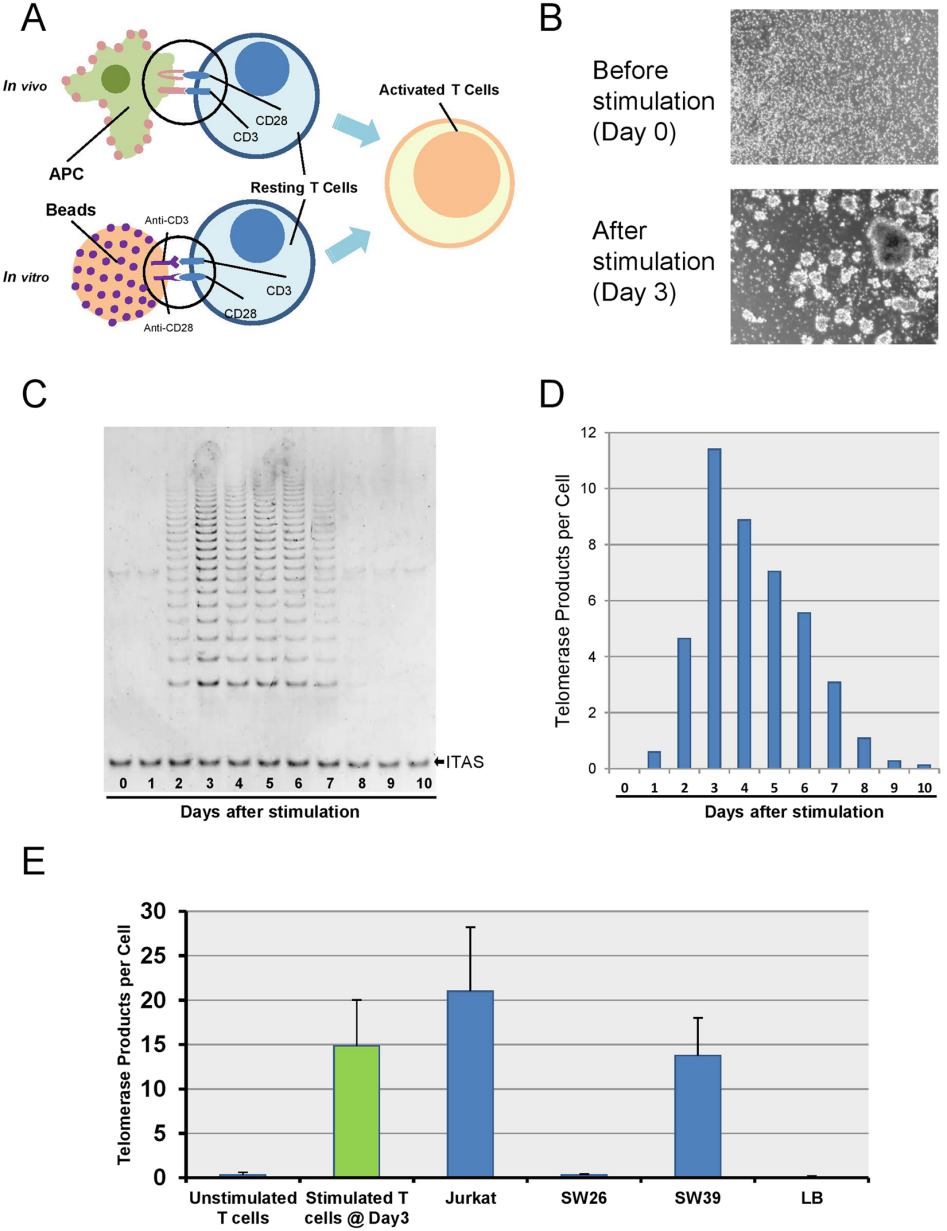
T lymphocytes stimulation model. (**A**) Bead activation mimics *in vivo* T cell activation from antigen-presenting cells (APC) by utilizing the two activation signals CD3 and CD28, bound to a 3D bead similar in size to the antigen-presenting cells. (**B**) Microscopic pictures of T lymphocytes before (day 0) & after (day 3) stimulation. (**C**,**D**) Transient telomerase activation in T cells measured by traditional gel-based TRAP assay and ddTRAP. (**E**) Comparison of telomerase activity among various cell types.

There are a variety of methods that can achieve similar outcomes for *in vitro* T cell stimulation, including concanavalin A (ConA)^17^, phytohaemagglutinin (PHA)^8, 18^, phorbol 12-myristate 13-acetate (PMA)/ ionomycin^18^, and anti-CD3/CD28. Among these, anti-CD3/CD28 is a cocktail of antibodies that binds to CD3 and CD28 on the surface of all T cells, triggering both signaling pathway I & II that promote T cells to proliferate^19^. As the specific binding to CD3 and CD28 surface proteins more closely mimics *in vivo* T cell activation from antigen-presenting cells (APC), we decided to use anti-CD3/CD28-coated beads to investigate telomere and telomerase dynamics in T cells during stimulation (Fig. 1A).

Previous reports have demonstrated that mitogen stimulated T lymphocytes transiently turn on telomerase activity for a short period of time (generally 5–10 days), even with continual mitogen stimulation^20^. We stimulated T cells with anti-CD3/CD28-coated magnetic beads, and observed that the cell population morphologically showed cell clustering/aggregation due to rapid cell division as soon as 2–3 days after stimulation (Fig. 1B). Consistent with the morphological changes, telomerase activity, as measured by the classic gel-based TRAP assay, is activated and peaks at approximately Day 3, then gradually drops back to baseline levels after ~10 days (Fig. 1C). This same phenotype has previously been reported by others^9, 11^ and we confirmed these results on 10 different individuals (Supplement 1). We compared the classic TRAP assay with ddTRAP assay, a novel droplet digital PCR TRAP protocol recently developed^16^. Even though measurements obtained by TRAP strongly correlated with those obtained by ddTRAP, the ddTRAP assay is more accurate and quantitative without the need for gel based quantification or internal PCR controls. (Fig. 1D). We next compared telomerase activity of stimulated T cells at day 3 (peak) with other cell lines by ddTRAP and observed that the peak magnitude of telomerase activity in stimulated T lymphocytes is comparable with that of several well characterized cancer cell lines (Fig. 1E). Both Jurkat and SW39 cells are capable of maintaining telomere length over time. This suggests that the levelf of telomerase activity detected in stimulated T cells is potentially sufficient to promote telomere length maintenance or at least slow down the rate of telomere erosion over multiple cell divisions.

### Telomerase activity is not required for the initial proliferation of T lymphocytes

**Figure 2:**
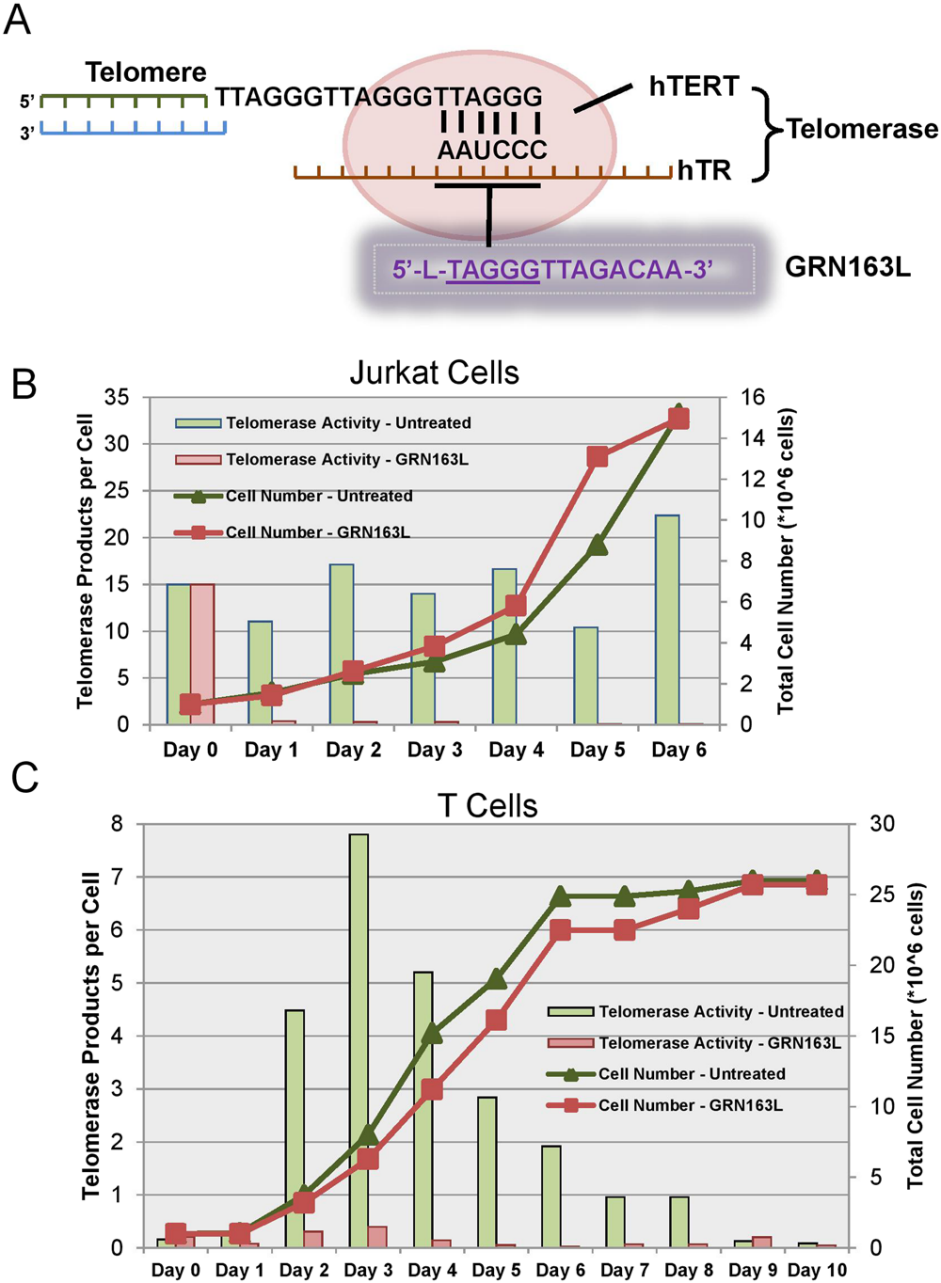
Inhibition of telomerase activity by GRN163L. (**A**) Schematic of GRN163L inhibition of telomerase activity. (**B**) Telomerase activity and cell growth curve of Jurkat cells treated with or without GRN163L. (**C**) Telomerase activity and cell growth curve of stimulated T cells treated with or without GRN163L.

Next, we tested whether telomerase activity, besides its potential function in maintaining telomeres, was necessary for the proliferation of T cells during stimulation. GRN163L (Imetelstat) is a 13-mer thio-phosphoramidate oligonucleotide that is complementary to the template region of telomerase RNA subunit (*hTR/hTERC*), and thus acts as a direct, competitive telomerase inhibitor (Fig. 2A). We first tested GRN163L’s effectiveness of blocking telomerase activity in Jurkat cells (an immortalized human T lymphocyte line). After treating Jurkat cells with GRN163L, telomerase activity was almost completely reduced within 24 hours, but the rate of cell proliferation was unaffected (Fig. 2B). This suggests that GRN163L can efficiently block telomerase activity in suspension cells and that telomerase activity is not required, at least initially, for the proliferation of Jurkat cancer cells. However, it was not known in normal T cells with regulated telomerase if GRN163L would affect stimulated T cell proliferation. Therefore, we tested GRN163L on stimulated T cells from healthy donors, and confirmed that untreated T cells activated telomerase to the expected levels whereas with GRN163L treatment telomerase activity was almost completely inhibited. This demonstrated that GRN163L can efficiently block telomerase activity in stimulated T cells (Fig. 2C). Importantly, we also observed that cells treated with GRN163L proliferated at very similar rates as the cells without GRN163L treatment (Fig. 2C). Our results provide further evidence that telomerase activity is not required for the short-term proliferation of normal T lymphocytes during stimulation at the population level, nor for the short-term proliferation of cancer cells (Jurkat).

### Lack of telomerase activity does not change the rate of telomere shortening in T cell populations during stimulation

**Figure 3:**
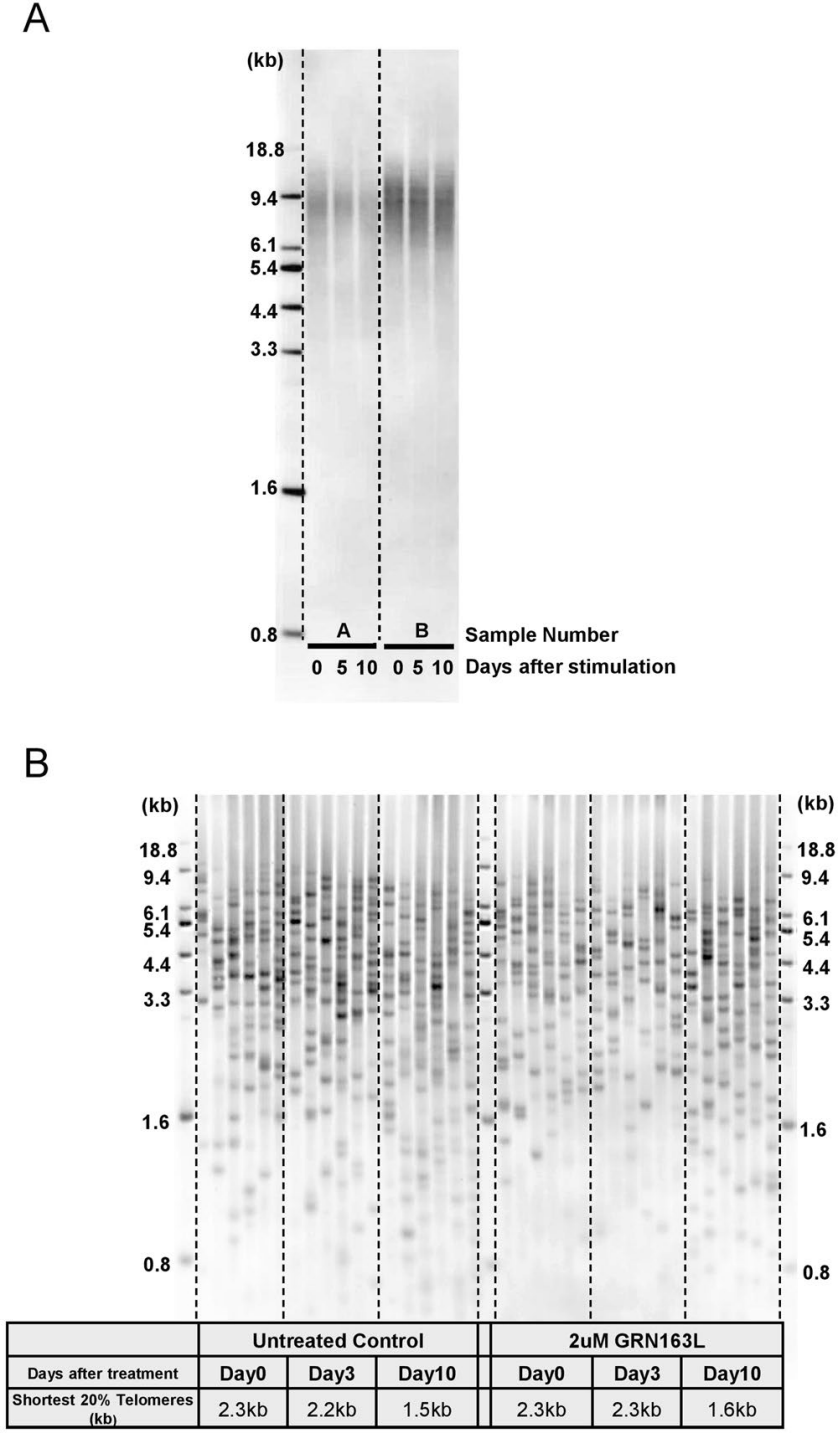
Measurement of telomere length during stimulation. (**A**) Telomere length during T cell stimulation, as detected by TRF assay. (**B**) Length of the shortest telomeres during T cell stimulation with or without GRN163L, as detected by TeSLA.

Although transient telomerase activation in stimulated T cells is widely believed to at least slow down the rate of telomere shortening during cell divisions, to the best of our knowledge this has never been demonstrated. It is possible that previous studies have been complicated by the lack of sensitive techniques to monitor subtle telomere length changes. The TRF (terminal restriction fragment lengths) assay is a widely used method to measure average telomere length by Southern blotting^21^. Using the TRF assay, we measured telomere length from stimulated T cells at day 0, day 5 and day 10, and could not detect telomere shortening over the ten day period (Fig. 3A). However, the TRF assay might not be sensitive enough to detect subtle changes in telomere dynamics, especially among the shortest telomeres which are known to be extremely important in determining the cellular replicative lifespan^22, 23^.

To further investigate telomere dynamics of T cells during stimulation, we developed a new technique. This assay measures the average as well as the percentage of all the shortest telomeres and thus can monitor small telomere changes with much higher sensitivity. In contrast with the results from TRF, the telomere shortest length assay results showed that before stimulation, the average length of the shortest 20% telomeres in the individual tested was 2.3 kb. Three days after stimulation, the length of the shortest 20% of telomeres was maintained at 2.2 kb, but after 10 days of stimulation this length decreased to 1.5 kb. (Fig. 3B, left panel). Significantly, when we blocked telomerase activity in stimulated T cells by GRN163L treatment and performed the telomere shortest length assay, we did not observe a significant increase in the load of shortest telomeres as initially expected (Fig. 3B, right panel). The average length of the shortest 20% telomeres in GRN163L treated T cells decreased at a similar rate as control stimulated cells from 2.3 kb to 1.6 kb. Thus, the lack of telomerase activity does not change the rate of telomere shortening in T cell heterogeneous populations during stimulation at least over the short-term.

We hypothesized that these observations were potentially due to high T cell heterogeneity and that only a small subset of stimulated T cells express robust telomerase activity. If correct, then only specific sub-populations of T cells maintain telomere length during antigen-induced cell divisions. When treated with GRN163L, we further predicted telomerase activity of this subset of T cells would be blocked, leading to more dramatic loss of telomeres during stimulation. However, when we performed the shortest telomere length assay on a heterogeneous population of T cells, the overall telomere loss of this subset of T cells would be diluted by the large population of T cells that did not activated telomerase. and would be masked when mixed populations of T cells are measured. Therefore, an in-depth investigation on some of the sub-populations of stimulated T cells became necessary to better understand telomerase and telomere dynamics during T cell stimulation.

### Only a subset of CD28+ T cells shows robust telomerase activity and is capable of maintaining telomere length during stimulation

**Figure 4:**
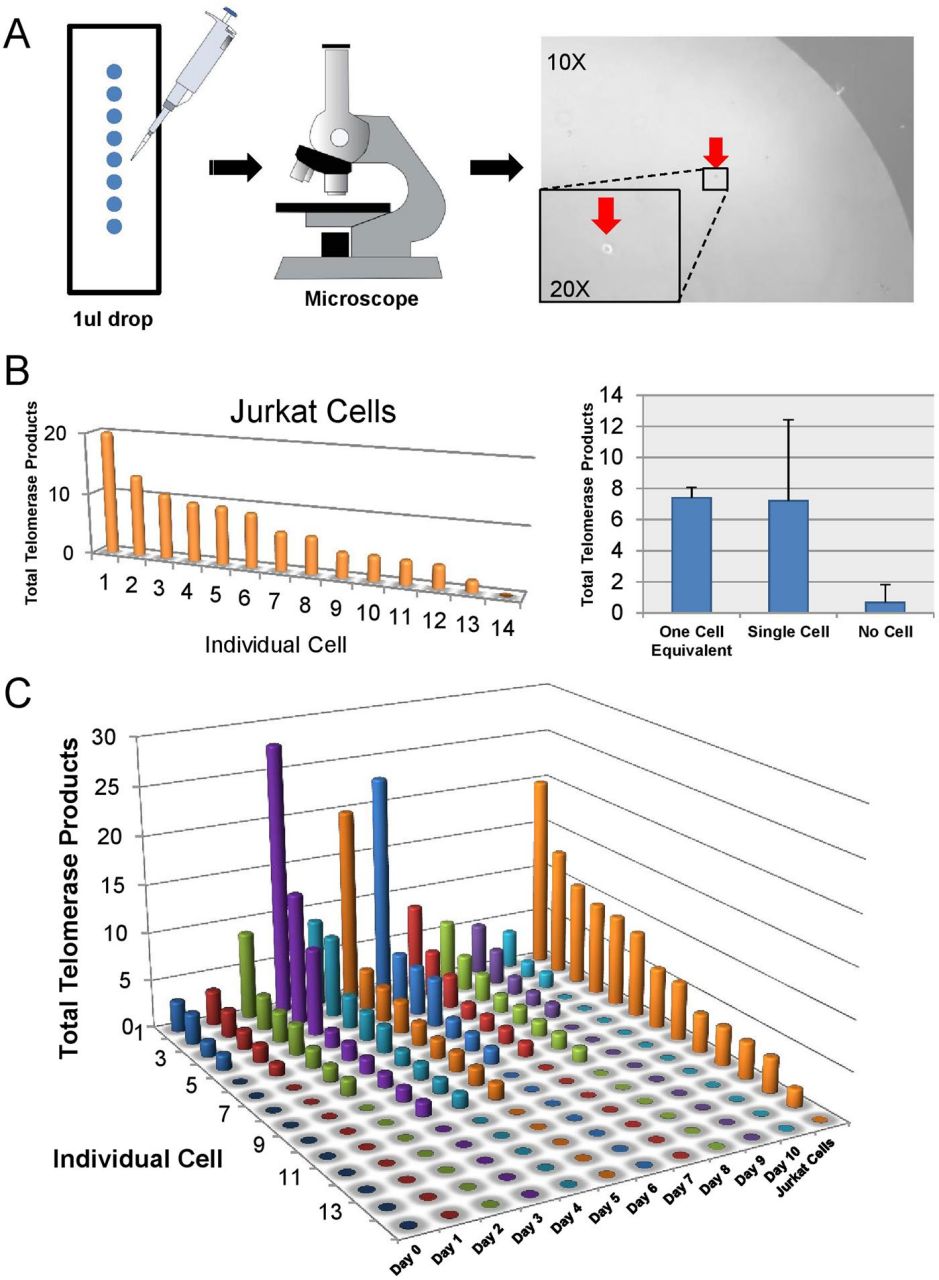
Measurement of telomerase activity at the single-cell level. (**A**) Schematic of single cell isolation procedure. (**B**) Single-cell ddTRAP performed on Jurkat cells. (**C**) Single-cell ddTRAP performed on stimulated T cells from Day 0 to Day 10.

Previous studies have demonstrated that at the single-cell level, T cells respond to antigen stimulation in an all-or-nothing manner, which is termed digital immune responses^24, 25^. However, it has not been determined if there is heterogeneity of telomerase activity at the single-cell level among different T cell sub-populations. We separated single cells by dilution plating aliquots (1 μL) drops on glass slides and visually confirmed the presence of single cells using a microscope (Fig. 4A). Next, we measured single cell telomerase activity by using a modified ddTRAP protocol^16^. We first tested the viability of single-cell ddTRAP in Jurkat cells, and found heterogeneity of telomerase activity in individual cells tested. As expected, the average telomerase activity of single cells was comparable to that of the whole population (Fig. 4B). We then applied single-cell ddTRAP on T cells from a healthy donor during a 10 day period of stimulation. Interestingly, only a small proportion of T cells (~14%) showed robust telomerase activity, while the vast majority exhibited little or no detectable telomerase (Fig. 4C). This heterogeneity of telomerase activity was confirmed on single cells from two other donors (Supplement Fig. 2A,B).

**Figure 5:**
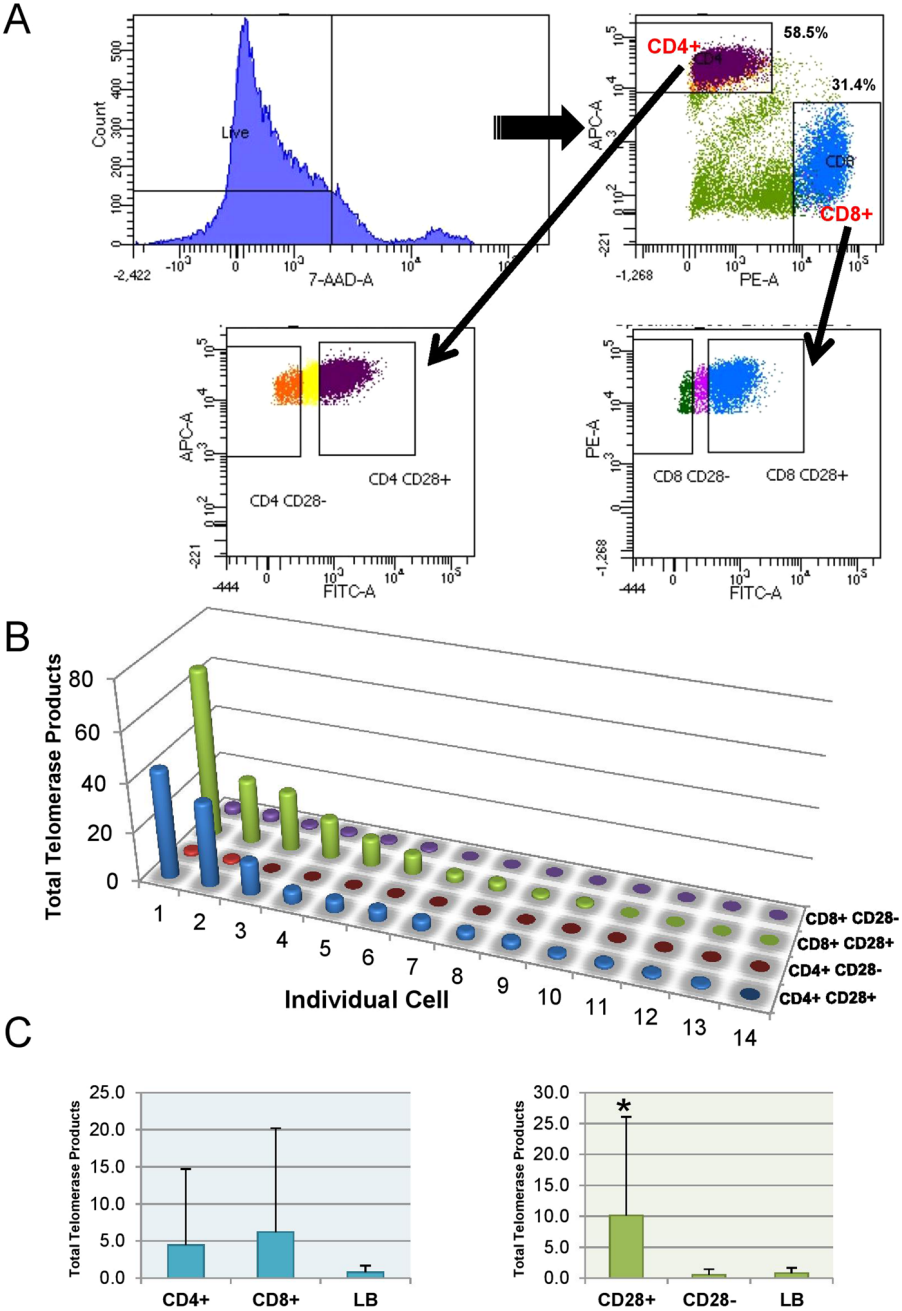
Telomerase activity in subsets of stimulated T cells. (**A**) Flow sorting strategy of stimulated T cells on Day 3 for separating four T cell subpopulations. (**B**) Single-cell ddTRAP on four subpopulations of T cells, i.e. CD4+ CD28+, CD4+ CD28−, CD8+ CD28+, CD8+ CD28−. (**C**) Cumulative analysis of (**B**).

It has been previously reported that the accumulation of CD28− T cells is associated with age-related decline of immune function^26^. There is also evidence showing that enriched populations of CD28+ T cells have higher levels of telomerase activity compared to the overall heterogeneous PBMCs population^27^. For example, when CD28 is ectopically expressed in CD8 T cells, the period of telomerase activity is extended^28^. Additionally, others reported differences in telomere length in resting T cells when comparing CD28+ and CD28− cells, with CD28+ showing slightly longer telomeres^14^. Considering the importance of CD28 in telomerase activation in T cells, we further separated the stimulated T cells at day 3 by flow sorting into four sub-populations: CD4+ CD28+, CD4+ CD28−, CD8+ CD28+, and CD8+ CD28− (Fig. 5A). Following single cell ddTRAP analysis, we found that only a subset of CD28+ cells, regardless of CD4+ or CD8+ expression, exhibited robust telomerase activity. In contrast, CD28− cells had little or no detectable telomerase (Fig. 5B,C). This was confirmed with two additional donors (Supplement 2C).

**Figure 6:**
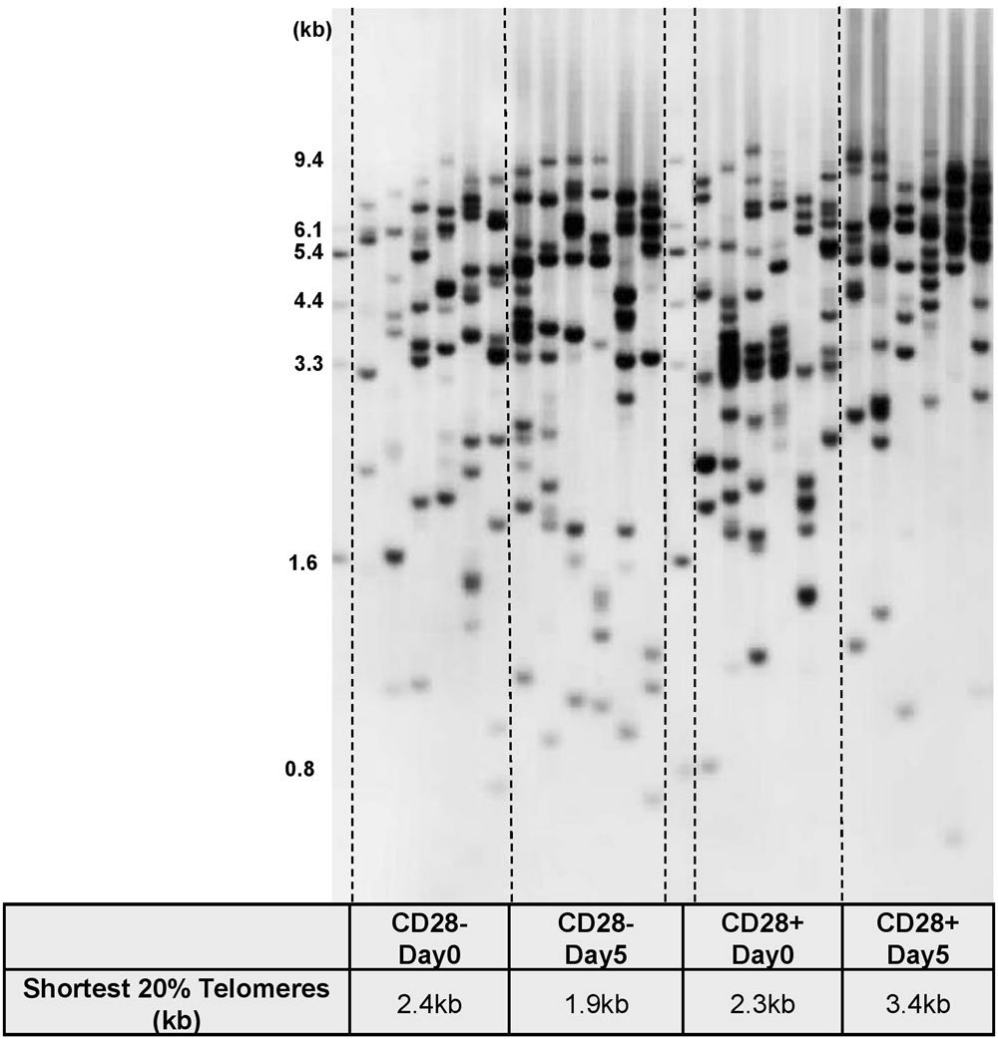
Length of the shortest telomeres in both CD28 positive and negative stimulated T cells.

We next examined the ability of CD28+ and CD28− T cells to maintain telomere length upon antigen-induced proliferation. Stimulated T cells at day 0 and day 5 were flow sorted into CD28+ and CD28−, and telomere length was measured. At Day 0, we found no significant difference in the length of the shortest 20% telomeres (2.4 kb vs 2.3 kb) between CD28− and CD28+ T cells, respectively (Fig. 6). However, after five days of stimulation, CD28+ cells exhibited lengthened telomeres whereas CD28− cells underwent progressive telomere shortening (3.4 kb vs 1.9 kb, respectively) (Fig. 6). These results can be interpreted to indicate that only a subset of CD28+ but not CD28− cells are capable of compensating for telomere loss through telomerase activity up-regulation during T cell stimulation.

## Discussion

Since transient telomerase activation in stimulated T cells was first described^9^, various studies have investigated the role of telomerase activity in cell proliferation and telomere maintenance during stimulation^10, 11^. In the short term, telomerase activity correlates with TERT mRNA splicing changes (Supplement Fig. 3), but the functional significance of this was unknown. Previous studies demonstrated that when *hTERT* was knocked down the proliferation capacity of T cells was not affected^13^, suggesting that the coupling of telomerase activation with cell proliferation was not functionally interrelated. In the current study, instead of knocking down hTERT, we used a small oligonucleotide GRN163L (Imetelstat) to treat T cells to minimize side effects while specifically and robustly blocking the function of telomerase without affecting its transcriptional or translational processes. When we uncoupled telomerase activity with cell proliferation by GRN163L, we demonstrated that telomerase activity is not required for T-cell proliferation, at least in the short term and on a population basis. However, our data are consistent with the idea that telomerase activity is required in the long-term for maintenance of telomeres in specific subsets of T lymphocytes to increase their sustained proliferation capacity. While this effect is not apparent from our short-term (10 day) culture experiments, it has previously been reported in the long-term culture of both T cells and acute myeloid leukemia (AML) cells treated with a dominant negative TERT^29, 30^.

Although there are previous studies describing telomerase and telomere dynamics in stimulated human T cells, most of the results were based on a mixture of population cell types. Since T cells are an extremely heterogeneous population of subtypes, the phenotype of the population may not reflect changes in specific cell types, especially in short term culture experiments. A previous study reported differences in telomere length between subsets of resting lymphocytes^31^. In stimulated T cells, it was reported that CD4+ T cells maintained telomerase activity at higher levels compared to CD8+ T cells after several rounds of stimulation^32^. Additionally, others have previously reported that telomerase activation does not ameliorate telomere erosion after stimulation of T cells^33^. In these studies all activated T cells in a heterogeneous population of cells had equal levels of telomerase activity, with no significant enrichment in telomerase activity detected among G1, S, and G2/M populations^10^.

Due to the lack of accurate and sensitive assays for both telomere length and telomerase activity, there have not been studies investigating telomere and telomerase dynamics within specific sub-populations of T cells during. By applying droplet digital PCR techniques to the classic telomerase TRAP assay, we have addressed the landscape of telomerase activity at the single cell level in stimulated T cells, and revealed that only a small subset of T cells from various healthy individuals show very strong telomerase activity upon stimulation. We further defined that this subset of T cells are largely restricted to CD28+ T cells. Previous studies have reported that the accumulation of CD28− T cells is one of the most prominent changes in the age-associated decline of immune function^26^. Also, sustained ectopic CD28+ expression in CD8+ T cells has been reported to delay replicative senescence, but this only indirectly reflects telomere length^28^. While there is a report that telomere elongation occurs in peripheral blood mononuclear cells (PBMCs) after 72 h stimulation in young aged groups (20–25 years old) using a qPCR telomere length assay^14^, we believe this might be due to the inaccuracy or large variation of qPCR measurements for average telomere lengths. In the current study, we used the classic TRF assay to measure average telomere length during stimulation and detected no changes, as would be expected for such a short period of cell proliferation. In contrast, the newly developed telomere shortest length assay revealed an accumulation of short telomeres during stimulation over the 10 day period. We further monitored telomere changes in sub-populations and provide more direct evidence that only the telomerase positive CD28+ T cells are capable of maintaining their telomere length thus avoiding the accumulation of short telomeres during stimulation. CD28 is a known activator of NF- kB that can transcriptionally upregulate telomerase levels. In addition, it has been shown that telomerase can directly regulate NF- kB-dependent transcription^34^. Thus a feed-forward regulation could potentially explain why telomerase activity is enriched in CD28+ T cells upon stimulation.

While telomerase activity is mainly restricted to CD28+ cells, we note that only a subset of CD28+ cells have detectable telomerase activity upon stimulation. One explanation is that telomerase activity fluctuates during cell cycle processes, but this is in contrast with previous findings that telomerase activity is not cell cycle phase restricted^10^. Another possible explanation could be that only further defined subsets of CD28+ cells are capable of activating telomerase and maintaining telomere length during stimulation. Memory T cells could represent one such subset of cells since they have been shown to possess the capacity to reconstitute the entire spectrum of effector T cell subsets, and participate in acute immune response to inflammation^35^. Furthermore, another report indicated that while average telomere length decreases in heterogeneous populations of naïve and memory T cells, a subset of naïveor memory T cells can maintain average telomere lengths when exposed to viral infections^36^. Our present studies indicate that further elucidation of a variety of distinct T cells showing robust telomerase activity upon stimulation may help elucidate the mechanism of T cell exhaustion with aging and age-related diseases.

## Materials and Methods

### Cell culture

Human peripheral blood samples were obtained from 10 healthy donors (20–35 years old) after informed consent and in accordance with the Institutional Review Board (IRB) at UT Southwestern Medical Center (IRB# CR00014523 / STU 042014-016). All subjects included in this study were nonsmokers with no history of alcohol abuse or drug consumption. Peripheral blood mononuclear cells (PBMCs) were isolated by centrifugation with Ficoll-Paque Plus (GE Healthcare) and T cells were further isolated by negative selection. T cells were cultured in RPMI+ GlutaMAX-I with 10% fetal bovine serum, 1% penicillin, streptomycin and amphotericin B and 10 ng/mL interleukin-2. Cells were stimulated 24 hours after isolation by adding Dynabeads Human T-Activator CD3/CD28 (Life Technologies) in a 1:1 ratio. The percent of live cells was determined every day by trypan blue exclusion using a TC20 Automated Cell Counter (BioRad). When the cell density reached ≥1.5 × 10^6^/mL, cells were diluted with fresh complete RPMI medium to a density of below 1.0 × 10^6^/mL. GRN163L (Imetelstat) was added 1 hour before stimulation at the concentration of 2 uM.

### Single-cell isolation and telomerase enzymatic activity assays

Telomerase activity was determined using the telomere repeat amplification protocol (TRAP), as previously described^7, 37^. The droplet-digital TRAP (ddTRAP) assay was performed as described^16^. Single-cell isolation was described previously^38^. In brief, cells were suspended and diluted in 1× PBS to 1000 or 2000 cells/mL, and then 1 μL aliquots were placed on a glass slide. Drops containing single cell were identified under the microscope, and then the visually confirmed 1 μL aliquots were mixed with 1 μL of ddTRAP lysis-extension buffer containing NP-40 buffer (1 mM Tris-HCl, pH8.0, 1 mM MgCl_2_, 1 mM EDTA 10% (vol/vol) glycerol, 150 mM NaCl, 5 mM β-mercaptoethanol, 0.1 mM AEBSF), 10× TRAP buffer (200 mM Tris-HCl, pH8.3, 15 mM MgCl2), 2.5 mM each dNTPs, 200 mM TS substrate, and 0.4 mg/mL BSA. The 2 μL lysis-extension mixture was pipetted into a PCR tube, placed on ice for at least 30 min incubation, followed by 40 min at 25 °C for telomerase extension reaction and 5 min at 95 °C for deactivation. After the extension reaction, the 2 μL lysis-extension mixture was ready for the standard ddTRAP protocol as previously described^16^.

### DNA extraction and telomeric measurement assays

Genomic DNA was extracted using the GentraPuregen DNA extraction kit (Qiagen) according to the manufacturer’s instructions. Each DNA sample was quantified on a Nanodrop (Thermo Scientific) for concentration/purity, and integrity of DNA was determined as previously indicated^39^. For DNA preparation from a small number of cells (i.e. 100 cells), T cells were resuspended in 1× PBS at the density of 1.0 × 10^5^/mL. Then 1 μL of cell suspension (100 cells equivalents) was mixed with 1 μL of NP-40 buffer (recipe described above) and incubated on ice for 30 min to lyse the cells. Then 1 μL of protease (QIAGEN) was added to the 2 μL mixture and incubate at 50 °C for 30 min to digest protein, followed by 70 °C for 15 min to deactivate the protease. The 3 μL mixture was directly used for the TeSLA (shortest telomere length) assay.

Terminal restriction fragment (TRF) assays were performed as previously described^21^.

The telomere shortest length analysis was performed as previously described^40^. In brief, each telomere overhang in the genomic DNA is ligated to telorettes by T4 DNA ligase (New England Biolabs). After ligation, the genomic DNA is digested by a series of restriction enzymes including CviAII, BfaI, NdeI, and MseI (New England Biolabs) to digest all DNA but not telomeres which do not have restriction site for these enzymes. The digested genomic DNA is next treated with Shrimp Alkaline Phosphatase (rSAP; New England Biolabs) to remove 5′ phosphate from each DNA fragment to improve the specificity of ligation between overhang adapters and genomic DNA fragments. For adaptor ligation,1 μM of AT adapter and 1 μM of TA adapter are mixed and ligated to the DNA fragment by T4 DNA ligase. After adapter ligation, multiple PCR reactions are performed (initial melt at 94 °C for 2 minutes followed by 26 cycles of 94 °C for 15 seconds, 60 °C for 30 seconds, and 72 °C for 15 minutes) using 2.5 units of FailSafe enzyme mix (Epicenter) with 1× FailSafe buffer H in 25 μl reaction containing 0.25 μM primers and 20–40 pg of ligated DNA. PCR products are resolved on a 0.85% agarose gel (1.5 V/cm for 19 hours). After gel electrophoresis, the Southern blot analysis to detect amplified telomeres is conducted as previously described^41^.

### Flow Cytometry

Surface expression of CD4, CD8, and CD28 was examined by flow cytometry. Cells were incubated with allophycocyanin (APC)-conjugated anti-CD4 (Invitrogen), PE-conjugated anti-CD8 (Invitrogen), and FITC-conjugated anti-CD28 (eBioscience) at 4 °C for 20 min, washed and fixed in PBS containing 2% 7-AAD. All samples were sorted and analyzed on a FACS Aria II SORP (5 lasers) flow cytometer. Fluorescence data from at least 50,000 cells were acquired. Data analysis was performed using Cell Quest Pro (BD Biosciences).

### Statistical Analysis

Mean values and standard deviations were calculated for each time-point. Significant difference was assessed by a two-tail Student’s *t* test, and a cutoff of *p* value < 0.05 was employed to determine significance.

## Electronic supplementary material

Supplementary Figures and Legends
