# Supplementary material for: The Maintenance of Telomere Length in CD28+ T Cells During T Lymphocyte Stimulation
Source: Sci Rep. 2017 Jul 28;7:6785. doi: 10.1038/s41598-017-05174-7 (PMC5533788; doi:10.1038/s41598-017-05174-7)

Supplementary Figures

The Maintenance of Telomere Length in CD28+ T Cells During T Lymphocyte Stimulation

Ejun (Elijah) Huang[1,2], Enzo Tedone[1,2], Ryan O'Hara[1], Crystal Cornelius[1], Tsung-Po Lai[1],Andrew Ludlow[1], Woodring E. Wright[1], Jerry W. Shay[1,*]

[1]Department of Cell Biology, UT Southwestern Medical Center, 5323 Harry Hines Blvd., Dallas TX 75390 U.S.A.,

[2]These authors contribute equally to this work.

*Corresponding Author:

Jerry W. Shay

Tel: 1- 214-648-4201

Fax: 1- 214-648-5814

Jerry.Shay@UTSouthwestern.edu

SUPPLEMENTAL FIGURE LEGENDS

Supplement Figure 1. Telomerase activity (ddTRAP) in PBMCs from 10 healthy donors stimulated over a period of ten days with anti-CD3 and anti-CD28 coated beads.

Supplement Figure 2. Single cell telomerase activity measurement in stimulated T cells from two additional healthy donors (A and B) showing the same trends as in Figure 4. (C) Telomerase activity in CD28 positive and CD28 negative T cells from 3 healthy donors.

Supplement Figure 3. (A). Diagram illustrating the exons and introns of human telomerase (hTERT). Functional telomerase is derived from the full length TERT transcript containing all 16 exons. Most of TERT transcripts are alternative spliced to inactive isoforms. (B). Using specific primers that amplify various TERT splicing forms, we profiled the full length and alternatively spliced variants of TERT in two healthy donors. In general, the TERT transcripts that incorporate both exons 7 and 8 peak between days 3-4 of stimulation and correlate with functional enzyme activity.

Supplementary Figure 1

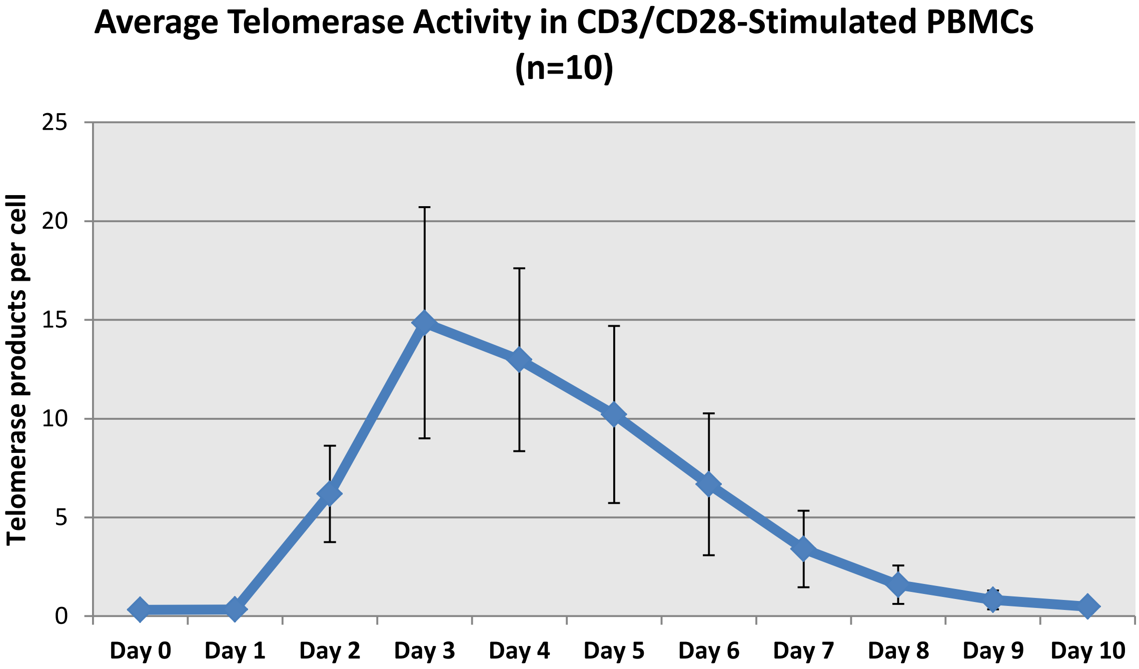

Supplementary Figure 2

A

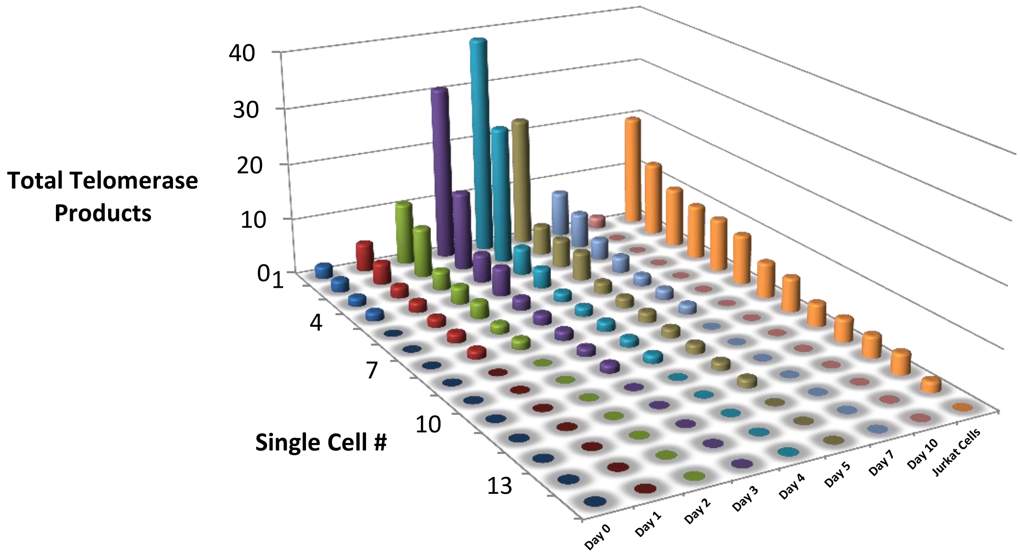

B

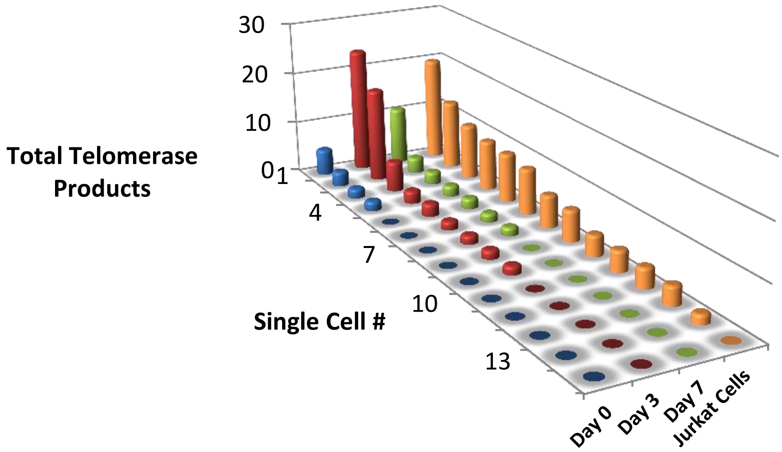

C

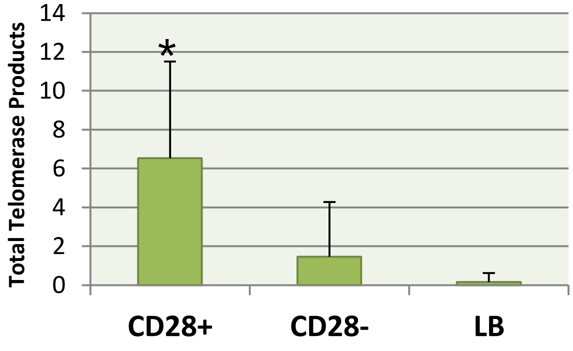

Supplementary Figure 3

A

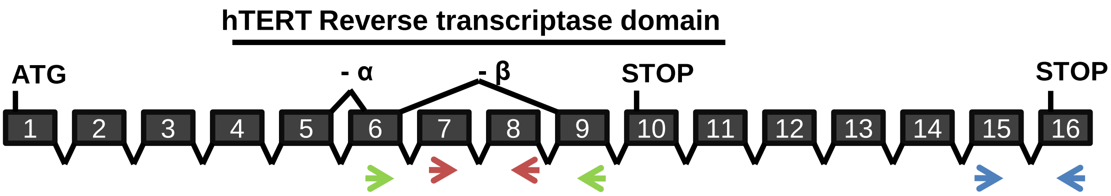

B

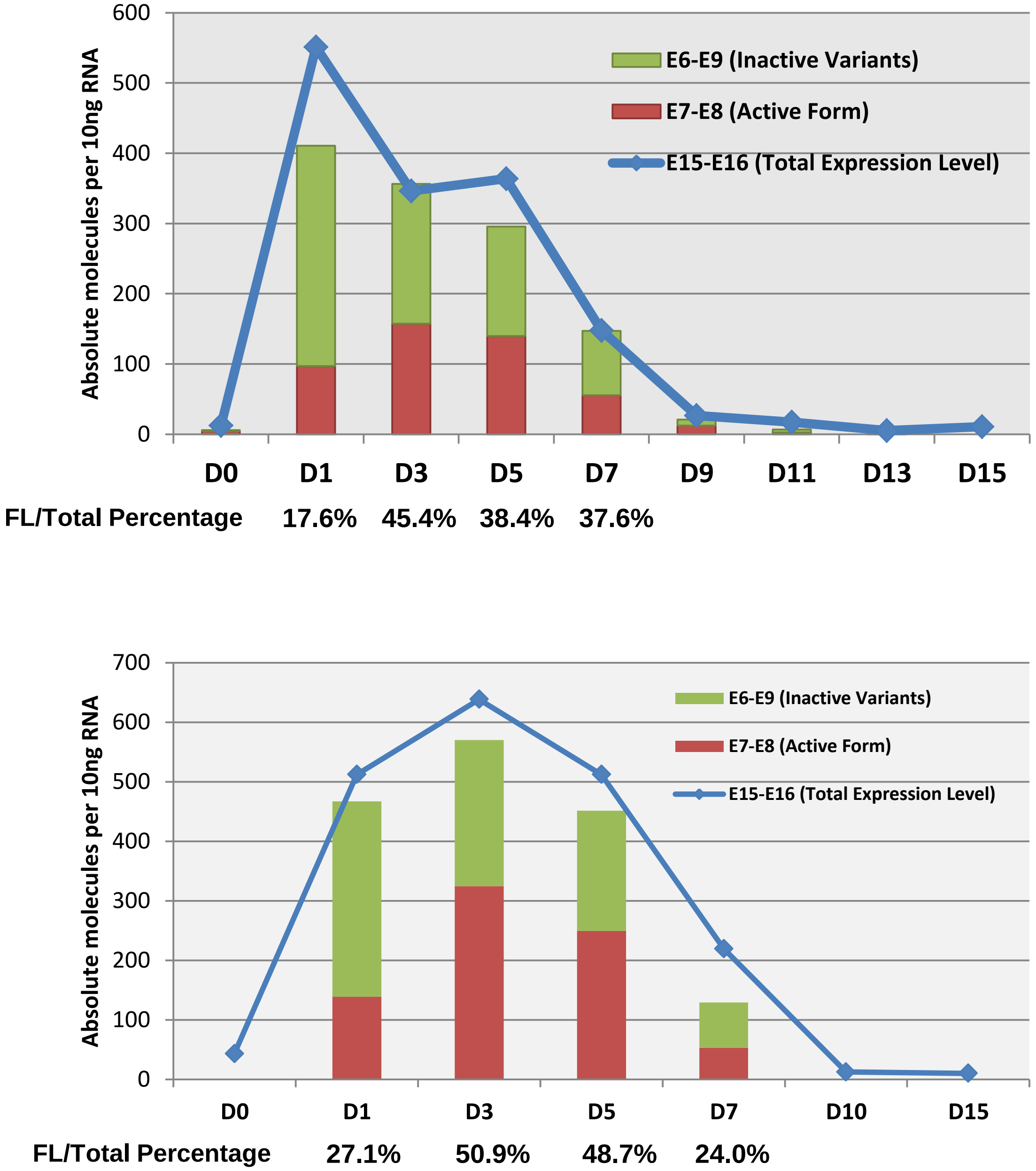

Supplement: Supplementary file 1 — Supplementary Figures and Legends [file 41598_2017_5174_MOESM1_ESM.pdf]
